# 2468 CTSI 500 Stars Initiative (CTSI of SE-Wisconsin)

**DOI:** 10.1017/cts.2018.201

**Published:** 2018-11-21

**Authors:** Doriel D. Ward, Orsolya Garrison, Chamia Gary, Memory Bacon, Tim Sobotka

**Affiliations:** The Medical College of Wisconsin

## Abstract

OBJECTIVES/SPECIFIC AIMS: Our Goal is to enroll 500 students over 10 years into the CTSI 500 Stars Initiative. Student family members and community members are essential to career achievement and success; as such, the program also engages student families, along with key community members, as part of an Advisory Group, throughout the entire student experience. Besides programmatic and planning activities, students, family, and community members participate in our CTSI Community Engagement Science Café monthly series, where students may also present on a number of research and health-related topics of interest. The Advisory Group meets every 3–4 months in ensuring continuous engagement and overall program success. METHODS/STUDY POPULATION: Our Initiative takes both direct and supportive roles in offering 2 educational and training pathways; namely, our Summer Internship Program (6–8 wk duration) and our Students Modeling a Research Topic (SMART) Year-round Education Program (usually offered in Fall and Spring academic semesters) for high school students only. In the SMART Teams program, we work with regional public and private school districts to train science teachers, and assist them in developing and/or enhancing their science curriculum, thus creating pathways towards careers in translational science settings. Our aim is that students who participate in the year-round program (along with additional students) subsequently participate in our summer program. Therefore, overall program engagement is continuous throughout the year. In Summer, 2017 we engaged with well-established regional partners and collaborators (CTSI affiliated numerous public school districts, and community-based organizations) to move the translational workforce along existing regional diversity education and training pipelines. A Kick-off event was held on June 15, 2107 and attended by students and family members. We offered 6–8 weeks of hands-on experiences working with faculty researcher mentors and their research teams conducting real-life studies, in addition to professional experiences in research “support” settings, as well as in the community. We also developed established a “Summer” SMART (Students Modeling a Research Topic) Teams Program and a Summer “Advanced” SMART Teams Program, where a number of students were placed at 2 CTSI partner and collaborator institutions. The primary goal of the SMART Teams experience is to introduce students to translational science by building upon laboratory research to better understand clinical and community impact of disease within a patient population. Overall, internship sites included research labs, protein modeling labs, numerous research support settings, clinical care settings, and community sites for those students who were interested in population health sciences. In addition, students were offered career enrichment and professional development lunch and learn sessions, career panel sessions presented by long term, expert professionals in various fields translational science, and confidence building and networking sessions. Students also participated in a community volunteer day activity, a trip to the Chicago Science Museum, and numerous CTSI engagement activities (Science Cafés, simulation lab tours, etc.). RESULTS/ANTICIPATED RESULTS: The 2018 year-round program will initiate in the Fall. Our 2017 Summer Internship Program received 192 students/trainees applications of whom 133 were underrepresented minorities (URMs). We enrolled 109 participants, including 83 URMs (84 high school students and 25 college students). A total of 53 Wisconsin high schools and 19 colleges and universities (local and out of state) participated. Students engaged in all activities as outlined in the Methods section. At the end of the summer program, students created and presented posters as part of the closing ceremony. Certificates of completion were given to the students by program leadership and the Al Hurvis/ADAMM leadership (program funding agency). Students wore white lab coats to create an atmosphere of cohesion and accomplishment. Parents and other family members attended the closing ceremony, demonstrating strong support for students and the program. Our anticipated results for CTSI 500 Stars Initiative is to increase diversity in the Translational Science Workforce via education and training of 500 high school and college students over 10 years. We will also remain engaged and track student’s various venues for at least 10 years to determine the outcome of their experiences towards careers in Translational Science settings. We will continue to engage community members and community-based organizations as collaborators and advisors to participate in every stage of our activities. Moreover, we plan to broaden our reach by establishing additional relationships with additional high schools and middle schools to further enhance the 500 Stars Initiative. In addition, we will develop metrics by which to measure the validity and success of our program. DISCUSSION/SIGNIFICANCE OF IMPACT: The aim of the CTSI 500 Stars Initiative is to provide real-life, practical experiences in translational science settings as a part of our efforts to train and cultivate the translational science workforce, while also engaging patients, families and community members in every phase of the translational process. Targeting under-represented minority students contributes towards increasing diversity in the workforce. It is also our hope that by increasing URMs in the workforce, there will be positive impact on communities of color, with respect to increasing participation in their health care decision making and in clinical/translational research; thus, ultimately leading to better health outcomes in the communities we live and serve. Our overall framework is to engage, educate, enrich, empower, elevate, enable students towards careers in clinical and translational settings.

